# *SHI*/*STY* Genes Affect Pre- and Post-meiotic Anther Processes in Auxin Sensing Domains in Arabidopsis

**DOI:** 10.3389/fpls.2018.00150

**Published:** 2018-02-14

**Authors:** Leandro H. Estornell, Katarina Landberg, Izabela Cierlik, Eva Sundberg

**Affiliations:** Department of Plant Biology, Swedish University of Agricultural Sciences, Uppsala BioCenter and Linnean Centre for Plant Biology in Uppsala, Uppsala, Sweden

**Keywords:** auxin, anther, pollen, SHI/STY, LRP1, PAO5, EOD3, PGL1

## Abstract

In flowering plants, mature sperm cells are enclosed in pollen grains formed in structures called anthers. Several cell layers surrounding the central sporogenous cells of the anther are essential for directing the developmental processes that lead to meiosis, pollen formation, and the subsequent pollen release. The specification and function of these tissues are regulated by a large number of genetic factors. Additionally, the plant hormone auxin has previously been shown to play important roles in the later phases of anther development. Using the *R2D2* auxin sensor system we here show that auxin is sensed also in the early phases of anther cell layer development, suggesting that spatiotemporal regulation of auxin levels is important for early anther morphogenesis. Members of the SHI/STY transcription factor family acting as direct regulators of *YUC* auxin biosynthesis genes have previously been demonstrated to affect early anther patterning. Using reporter constructs we show that *SHI*/*STY* genes are dynamically active throughout anther development and their expression overlaps with those of three additional downstream targets, *PAO5, EOD3* and *PGL1*. Characterization of anthers carrying mutations in five *SHI*/*STY* genes clearly suggests that SHI/STY transcription factors affect anther organ identity. In addition, their activity is important to repress periclinal cell divisions as well as premature entrance into programmed cell death and cell wall lignification, which directly influences the timing of anther dehiscence and the pollen viability. The SHI/STY proteins also prevent premature pollen germination suggesting that they may play a role in the induction or maintenance of pollen dormancy.

## Introduction

**FIGURE 1:**
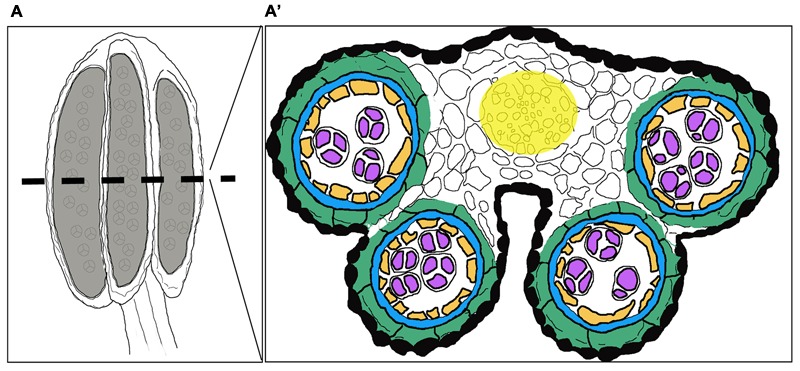
Overview of a stage 7 stamen and its internal composition. Schematic drawings of **(A)** the stamen in a longitudinal section and **(A′)** a cross-section depicting the internal structure of the anther with cell layer arrangement highlighted in different colors. Black: Epidermis; Green: Endothecium; Blue: Middle layer; Orange: Tapetum; Purple: Gametophyte; White: Connective tissue; Yellow: Procambium.

Stamens, the male reproductive organs in flowering plants, carry an apical anther and a basal filament transmitting water and nutrients from the shoot to the anther. The anthers of the model species *Arabidopsis thaliana* are four lobed structures, where each lobe forms chambers called locules, separated by connective tissue (**Figure 1**). Each locule consists of centrally positioned sporogenous cells producing microspores upon meiosis. The sporogenous tissue is surrounded by four somatic cell layers that contribute to the production and release of mature pollen grains carrying male gametes: the outermost cell layer, the epidermis; the endothecium, which upon lignification is instrumental in anther opening and pollen release; the middle layer (ML), which is important for pollen development, and finally the innermost layer, the tapetum, which is required for nourishment and development of the pollen (Esau, 1977; Pacini et al., 1985; Goldberg et al., 1993; Scott et al., 2004; Wilson et al., 2011; Fernández Gómez et al., 2015). During later stages of development specific cell types enter programmed cell death (PCD), while others go through modifications allowing pollen dispersal. For a recent review of anther development and the different tissue layers, see Fernández Gómez et al. (2015).

The specification, differentiation and function of the cell layers of the anther locules are regulated by a large number of identified genetic factors (for review see Kelliher et al., 2014; Walbot and Egger, 2016). In addition, the plant hormone auxin has been suggested to play important roles not just for stamen primordia initiation, but also for developmental processes in several of the anther cell layers (Cardarelli and Cecchetti, 2014).

Although *YUCCA* (*YUC*) auxin biosynthesis genes as well as *ABCB/PGP* genes encoding auxin efflux carriers are expressed in early stamen primordia (anther stages 1–5; stages according to Sanders et al., 1999) as well as in tapetum and pollen mother cells (PMCs) entering into meiosis (anther stage 6), *DR5pro:GUS* activity in anthers could not be detected until anther stage 8, when microspores are released (Cheng et al., 2006; Cecchetti et al., 2008, 2015). Thus, the auxin response profile, and as a consequence, the function of auxin during the pre-meiotic phases of anther development, are so far poorly understood. Still, some data clearly points to specific roles of auxin during pre-meiotic phases. Lines carrying mutations in both *ABCB1* and *ABCB19* show unsynchronized and precocious meiosis (Cecchetti et al., 2015).

More data is available regarding auxin dynamics at post-meoitic stages. At anther stage 8, *DR5pro:GUS* is mainly active in the endothecium and ML, whereas both *ABCB1pro:GUS* and *ABCB19pro:GUS* are active only in the tapetum, microspores and procambium (Cecchetti et al., 2008, 2015, 2017). Based on this, and results from NPA treatment, which prevents auxin efflux, Cecchetti et al. (2017) suggested that auxin at this stage flows from the tapetum to the ML, and that the auxin peak in this layer is important for coordinated pollen maturation and anther dehiscence. At the following stage (9) auxin responses could be detected in the endothecium, tapetum and in the microspores, and at stage 10, when degeneration of the tapetum is initiated, *DR5pro:GUS* was mainly detected in the tapetum and the procambium revealing a highly dynamic distribution of auxin signaling peaks (Cecchetti et al., 2008, 2017). Lines carrying mutations in the four *TIR*/*AFB* genes encoding nuclear auxin receptors show premature pollen maturation and germination, as well as premature endothecium lignification and stomium breakeage, leading to early anther dehiscence and pollen release (Cecchetti et al., 2008, 2013). This phenotype suggests that auxin signaling is important to repress precocious entering into subsequent developmental steps in several anther cell layers. In addition, the *abcb1 abcb19* double mutant anthers over-proliferate tapetum cells, and show enhanced septum lignification, implicating that auxin transport is instrumental in the control of cell division and the level of lignification (Cecchetti et al., 2015).

Members of the Arabidopsis SHI/STY family affect the identity and early patterning of stamen primordia (Kuusk et al., 2002, 2006). They have highly redundant functions during plant development, and act as DNA-binding transcriptional activators directly binding members of the *YUC* family (Kuusk et al., 2002, 2006; Sohlberg et al., 2006; Eklund et al., 2010). Overexpression of individual SHI/STY members results in a delay in dehiscence and consequently reduced pollen release (Kim et al., 2010) which suggests that SHI/STY proteins may play important roles throughout anther development, from early primordial establishment to maturation. Their function during anther development could in part be mediated through regulation of auxin biosynthesis. However, STY1 also directly regulates a large number of additional genes. The transcription of e.g., *POLYGALACTURONASE LIKE 1* (*PGL1*), *CYTOCHROME P450 78A6*/*ENHANCER OF DA1-1* (*CYP78A6*/*EOD3*) and *POLIAMINE OXIDASE/DEHYDROGENASE 5* (*PAO5*) is significantly upregulated by STY1-GR nuclear translocation in the presence of the translational inhibitor cycloheximide (CHX), and downregulated in floral buds of a quintuple *shi/sty* mutant, strongly implicating that they are direct STY1 targets (Ståldal et al., 2012). PGL1 belongs to a group of cell-wall modifying pectin lyases and is highly expressed during floral abscission and in stigmas (Kim et al., 2006; Cao, 2012), while EOD3 is a member of the CYP78A sub-family of cytochrome P450 related proteins. EOD3 together with its paralog CYP78A9 promotes ovule integument growth, thus controlling seed size, and is expressed in leaves and carpels suggesting that it may promote organ growth (Fang et al., 2012). *PAO5* encodes a cytosolic polyamine dehydrogenase that catalyzes the conversion of spermine (Spm) and thermospermine (T-Spm) to spermidine (Spd) (Ahou et al., 2014; Kim et al., 2014). PAO5 has been implicated in root xylem differentiation (Alabdallah et al., 2017) and is expressed in tapetal cells and at the anther-filament junction (Fincato et al., 2012).

Here we aimed to study the function of SHI/STY members and their putative targets *EOD3, PAO5* and *PGL1* in anther morphogenesis. We have characterized their expression domains throughout anther development as well as the anther morphology of a quintuple *SHI*/*STY*-family mutant line (Kuusk et al., 2006). We also used the ARF-independent *R2D2* auxin sensor system (Liao et al., 2015) to characterize auxin responses during pre-meiotic anther development. Our results reveal that auxin sensing is spatiotemporally controlled throughout anther development, including the pre-meiotic phase, and that *SHI/STY* genes are important in the control of processes also regulated by auxin, as well as in processes not yet linked to auxin action.

## Materials and Methods

### Plant Material and Growth Conditions

All lines used in this study are in the *Arabidopsis thaliana* Columbia ecotype background unless otherwise stated. The following lines have been described previously: *EOD3pro:GUS* (Fang et al., 2012), *PAO5pro:GUS* (Fincato et al., 2012), *SHIpro:GUS* (Fridborg et al., 2001), *STY1pro:GUS* and *STY2pro:GUS* (Kuusk et al., 2002), *SRS5pro:GUS* and *sty1-1 sty2-1, shi-3 lrp1 srs5-1* (Kuusk et al., 2006), *DR5rev:GFP* (Friml et al., 2003) and *R2D2* (Liao et al., 2015). Seeds were surface-sterilized as previously described (Fridborg et al., 1999), cold-treated for 2–3 days before germination, and cultured in cool white fluorescent light at 20–22°C under long-day photoperiod (16 h light, 8 h darkness). Samples were collected from the primary inflorescence around 4 weeks after transplantation to soil.

### Reporter Gene Constructs and Generation of Transgenic Lines

For GUS reporter gene constructs, genomic DNA was isolated from *A. thaliana* floral buds and used to amplify sequences immediately upstream of the translational start site from *PGL1* (2.6 kb), *LRP1* (3 kb), *SRS6* (0.44 kb) and *SRS7* (1.9 kb) using the Phusion High-Fidelity DNA Polymerase (Thermo Scientific), and gene specific oligonucleotides (Supplementary Table 1). Purified fragments were transferred by Gateway recombination into pGWB3 (Nakagawa et al., 2007) (Supplementary Figure 1). Restriction analysis and sequencing were used to confirm the vector-insert joining regions. Subsequently the plasmids were transferred to *Rhizobium radiobacter* strain C58C1 by the freeze-thaw method and transformed to Arabidopsis as described (Bechtold et al., 1993). Homozygous lines were selected using the kanamycin resistance marker. Genotyping was performed by PCR using leaf DNA extracts (Edwards et al., 1991) and the oligos in Supplementary Table 1.

### Sample Preparations

For GUS staining, at least ten inflorescences from three independent lines were collected in ice-cold 90% (v/v) acetone. Samples were vacuum infiltrated twice for 10 min, first with x-Gluc-devoid GUS-staining solution (De Block and Debrouwer, 1992) and then with GUS staining solution in which they were further incubated for 24 h at 37°C in the dark. Samples were destained through a grade-increasing ethanol series (20, 35, 50, 70%, 30 min incubation each) and stored in 70% ethanol at 4°C according to Jefferson et al. (1987).

For phenotypic characterization of the quintuple *shi*/*sty* mutant anthers, at least 10 inflorescences each of the mutant line and of wild type Columbia and Landsberg erecta (Ler) were fixed in FAA (3.7% v/v formaldehyde, 50% v/v ethanol, 5% v/v acetic acid) and incubated overnight at 4°C and thereafter dehydrated by incubation in 20% increasing ethanol series (30 min each and starting with 10% ethanol). Samples were stored in 70% ethanol at 4°C.

For sectioning of quintuple mutant, Columbia and GUS stained anthers, the fixed inflorescences were embedded in GMA (Leica Historesin Embedding Kit, Leica Biosystems Nussloch GmbH, Heidelberg) following the manufacturer’s protocol with some modifications according to Paiva et al., 2011. Using a Microm HM 355S microtome, the quintuple mutant samples were cut into 3 μm thick cross-sections, which were stained with 0.05% (w/v, 0.1 M sodium acetate pH 4.7) toluidine blue O (Sigma, St. Louis, MO, United States). GUS stained samples were cut into 8 μm transverse sections.

For confocal imaging of the spatiotemporal expression of the *R2D2* and *DR5rev:GFP* fluorophores, inflorescences were collected and treated according to Larsson et al. (2014). After washing the samples with PBS three times, individual flowers were dissected and stamens were mounted in 30% glycerol.

For nuclear staining of quintuple mutant anther sections, slides with 3 μm thick GMA embedded inflorescence cross-sections were covered by a solution of 1 μg/ml DAPI in 50 mM Tris, pH 8.0 (Kim et al., 2010). Slides were mounted and incubated for 1 h in darkness before image visualization.

For TUNEL assays at least 10 inflorescences from different plants of L*er* and quintuple mutant were first vacuum infiltrated for 20 min and incubated for 24 h at 4°C in a fixative solution containing 4% (w/v) paraformaldehyde in PBS (pH 7.2). Samples were washed in PBS at room temperature and dehydrated in a graded ethanol series (seven steps of 10% increments, 30 min each starting with 10% ethanol). Samples were cleared by applying successive steps (1 h at RT) of increase histoclear concentration: 25, 50, 75%, and by incubating them 3 times in 100% histoclear for 30 min each. Samples were paraffin embedded to make 8 μm sections, which were attached on polylysine-coated slides. The day of the TUNEL assay, slides were de-paraffined with histoclear, incubated in absolute ethanol for 5 min and hydrated through a series of four steps of 3 min of 20% decreasing ethanol. Slides were washed 2 × 5 min with 0.85% NaCl and PBS. Proteinase k treatment and *in situ* end labeling of nuclear DNA fragmentation was carried out using a TUNEL apoptosis kit (DeadEnd_ Fluorometric TUNEL System, Promega) and following the manufacturer’s instructions. As positive controls we used L*er* and quintuple slides treated with 10 units/mL of RNase-free DNase I (Thermo Fisher Scientific, Inc., Waltham, MA, United States) for 10 min at room temperature prior to the TUNEL labeling reaction. Positive controls were washed in separate jars following the TUNEL apoptosis kit protocol. Both samples and positive controls were counterstained with a solution of DAPI (1 μg/mL) in VECTASHIELD antifading agent (Vector Laboratories, Burlingame, CA, United States) and kept 2 h at 4°C in the dark prior observation. The positive controls behaved as expected.

For Alexander staining assay, pollen from flowers at anthesis was collected from 10 different inflorescences of each of Col-0 and L*er*. The pollen was spread on slides and covered with 30 μL of Alexander staining solution (Alexander, 1969). In order to assess pollen viability within closed anthers, stage 12 *shi*/*sty* anthers from 6 different inflorescences were collected and placed on slides containing drops of the Alexander solution. The slides were covered and incubated 4 h on a 50°C surface prior to observation. Anthers of the quintuple *shi/sty* mutant were classified based on phenotypic severity and their estimated percentage of viable pollen content (less than 50% or at least 50%). The number of anthers falling into each of the two viability categories was counted for each phenotypic class. The assay was repeated for a total of 3 independent experiments.

For pollen germination assays, pollen from flowers at anthesis was collected from 10 different inflorescences of each of the quintuple *shi/sty* mutant, Col-0 and L*er*. Three independent experiments were performed. Inflorescences were opened with forceps and pollen was extracted by brushing the stamens on slides containing agarized medium prepared according to Li et al. (1999). The slides were incubated at 22°C for 48 h inside a humidified chamber similar to the one described in Johnson-Brousseau and McCormick (2004). Samples were visualized after 24 and 48 h incubation times. To determine germination ratios, both germinating (pollen tube length > 10 μm) and non-germinating mature pollen were counted. Irregular-shaped smaller grains and collapsed bodies corresponding to arrested microspores were not included in the analysis.

### Microscopy and Imaging

GUS, Alexander, DAPI, TUNEL and germination assays were analyzed using a Zeiss Axioplan Fluorescence microscope (Carl Zeiss, Oberkochen, Germany) with a DFC295 camera for imaging. In order to get an estimation of GUS intensity, we determined the decrease in brightness in the Red channel according to Pozhvanov and Medvedev (2008). Areas corresponding to the different cell layers were selected, the cell number counted and Red channel mean and maximum intensity values determined for each area to calculate DR and its associated error. For TUNEL and DAPI assays two emission filters were used, 520 nm for fluorescein detection, and 420 for DAPI detection.

Confocal laser-scanning micrographs of *R2D2* and *DR5rev:GFP* anthers were obtained using a Zeiss 780 Inverted Axio Observer with a supersensitive GaASp detector and a C-Apochromat water immersion objective with a 1.2 numerical aperture. Confocal scans were carried out using a pinhole equivalent to 1 Airy unit and a two-track scanning strategy was followed to avoid cross-talk between fluorophores. Venus florescence was excited at 514 nm and detected at 518–553 nm, tdTomato was excited at 561 nm and detected at 566–637 nm, eGFP was excited at 488 nm and detected at 493–598 nm and Chlorophyll B was excited at 633 nm and detected at 638-721 nm. Both single images and z-stacks were captured and processed with ZEN software (Carl Zeiss, Oberkochen, Germany).

Image quantification, counting and processing were made using ImageJ (Schneider et al., 2012), and Photoshop CC was used to merge images corresponding to different channels.

### Statistical Analysis

Statistical analysis was carried out using the R software and R packages agricolae (de Mendiburu, 2016) and dunn.test (Dinno, 2017).

## Results

### *SHI/STY* Gene Activity Partially Overlap during Stamen Patterning, Pollen Development, Tapetum Degradation and Anther Dehiscence

**FIGURE 2:**
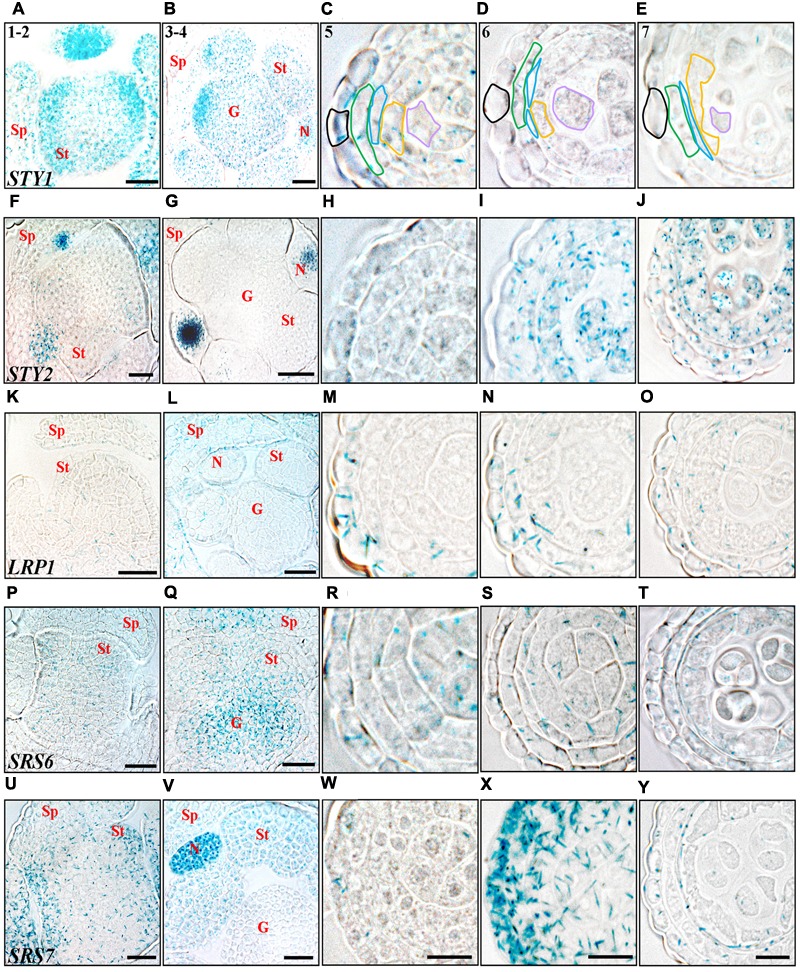
Histochemical analyses of *SHI/STY* promoter activity through early stages of anther development. **(A–E)** *STY1pro:GUS*. **(F–J)** *STY2pro:GUS.* **(K–O)** *LRP1pro:GUS*. **(P–T)** *SRS6pro:GUS*. **(U–Y)** *SRS7pro:GUS*. **(A,F,K,P,U)** Stage 1–2, emergence of stamen primordia. **(B,G,L,Q,V)** Stage 3–4, four-lobed pattern generated. **(C,H,M,R,W)** Stage 5, anther cell types distinguishable. **(D,I,N,S,X)** Stage 6, PMCs enter meiosis. **(E,J,O,T,Y)** Stage 7, tetrads are formed. Promoter activity is visible as blue staining in cross-sections of floral buds (stages 1–2 and 3–4) and microsporangia (stages 5–7). Letters indicate floral parts or their primordial equivalents, Sp, Sepal; St, Stamen; G, Gynoecium; N, Nectary. Anther stages are indicated with numbers and individual cell-boundaries have been highlighted in *STY1pro:GUS* microsporangia cross-sections, Black: Epidermis; Green: Endothecium; Blue: Middle layer; Orange: Tapetum; Purple: Gametophyte. Bars in stages 1–2 and 3–4 = 20 μm. Bars in stages 5–7 = 10 μm. Microsporangia pictures of each stage are at the same magnification and representative bars are only shown for *SRS7pro:GUS*.

The anther stage characterization made by Sanders et al. (1999) has been used to define the developmental stages studied. At the emergence of stamen primordia (anther stage 1–2), *STY1, SHI* and *SRS7* activity is strong throughout the primordial tissue and the activity remains high at stage 3–4, during which microsporangia cell types are determined (**Figure 2** and Supplementary Figures 2, 3A,K). This suggests that *SHI*/*STY* genes are important during primordia establishment and stamen patterning. The initial *SHI/STY* activity peak diminishes and at stage 5 the expression of several of the *SHI*/*STY* genes is low in all internal cell layers of the anther (**Figure 2**). However, *SHI* and *STY1* expression remains strong in the tip of the anther, and additional apical tip expression of *STY2* and *SRS5* is activated at stage 5 (Supplementary Figures 3B,G,L,R). The apical expression of all four genes remains high during development, and at later stages *SRS7* expression is also activated (Supplementary Figures 3CC,DD).

**FIGURE 3:**
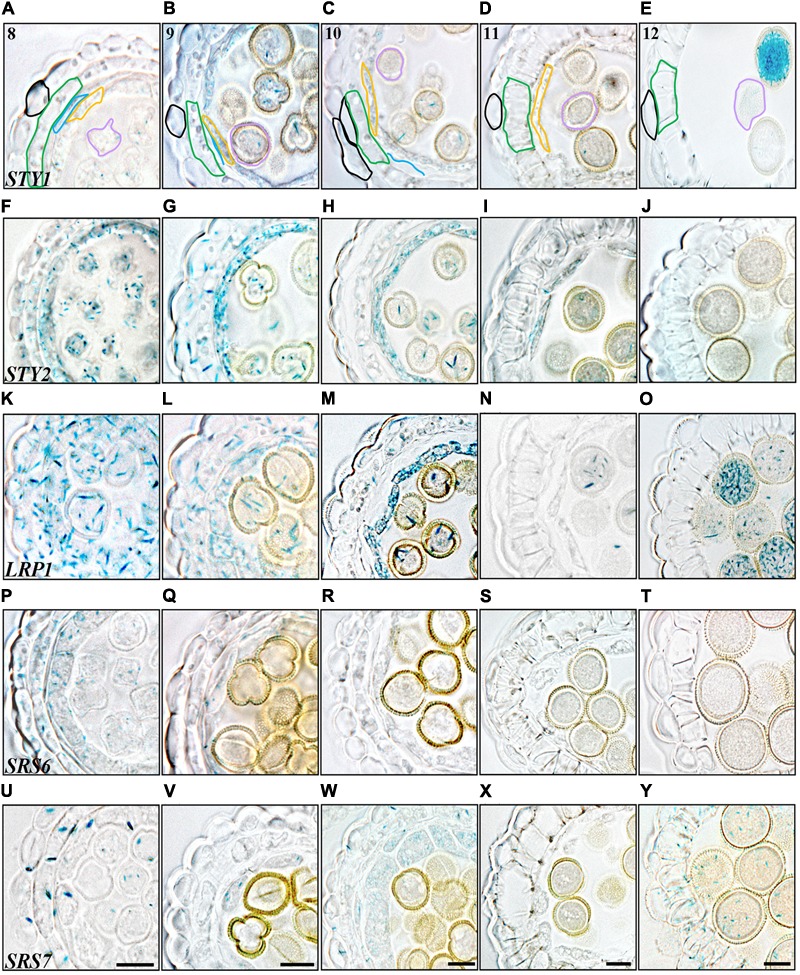
Histochemical analyses of *SHI/STY* promoter activity through late stages of anther development. **(A–E)** *STY1pro:GUS* staining. **(F–J)** *STY2pro:GUS* staining. **(K–O)** *LRP1pro:GUS* staining, **(P–T)** *SRS6pro:GUS* staining. **(U–Y)** *SRS7pro:GUS* staining. **(A,F,K,P,U)** Stage 8, microspores released. **(B,G,L,Q,V)** Stage 9, microspores with exine wall. **(C,H,M,R,W)** Stage 10, tapetal degeneration initiated and microspores binuclear. **(D,I,N,S,X)** Stage 11, lignified endothecium. **(E,J,O,T,Y)** Stage 12, tapetum disappearance and pollen trinuclear. Promoter activity is visible as blue staining in cross-sections of microsporangia. Anther stages are indicated with numbers and individual cell-boundaries have been highlighted in *STY1pro:GUS* cross-sections, Black: Epidermis; Green: Endothecium; Blue: Middle layer; Orange: Tapetum; Purple: Gametophyte. Bars = 10 μm. All pictures of each stage are at the same magnification and representative bars are shown for *SRS7pro:GUS*.

*STY2* and *LRP1* are strongly activated in the pollen mother cells (PMC) and in the tapetum cells at stage 6 and 8, respectively, and remain active in both these cell types until stage 11 (**Figures 2, 3**). During this time the PMC enter meiosis, form microspore tetrads that are released at stage 8, and then further differentiate into three-celled pollen grains during stage 9–12. The tapetum layer contributes to pollen development by releasing material needed for the pollen exine wall, under a process of degradation via PCD (Quilichini et al., 2015). In addition *STY1* and *SRS7* also becomes active in the tapetum from stage 9 (**Figures 3B,V** and Supplementary Figures 2A,B), suggesting that *SHI/STY* genes are important for proper tapetum development and function, as well as for the degradation of this cell layer. Interestingly a second burst of *STY1* and *LRP1* activity is apparent in pollen grains at stages 12–13 (**Figures 3E,O** and Supplementary Figures 2, 3O,P,W–Y), indicating that the SHI/STY family is not only important during pollen development, but could also have a function at maturity.

In the three outermost cell layers of the anther, the epidermis, endothecium and ML, a low level of *STY2, LRP1* and *SRS6* expression as well as a strong transient activity of *SRS7* is detected at stage 6 (**Figures 2I,N,S,X**). The *SRS7* activity decline again, but clearly remains in the ML at stage 7 (3/5 of samples analyzed) (**Figure 2Y**). At stage 12, only *SRS7* expression is detected in the epidermis and some areas of the lignified endothecium (**Figure 3Y** and Supplementary Figures 2, 3EE). In the final stages of stamen maturation the septum is lysed to produce a bi-locular anther and upon dehydration the stomium opens. *SRS7* could be involved in the processes leading to opening of the stomium and pollen release. Essential for self-pollination during anthesis is a quick growth of the stamen filament at the last stages (Goldberg et al., 1993; Scott et al., 2004). Of the *SHI*/*STY* genes analyzed, *LRP1, SRS6* and *SRS7* show a late expression in the filament vasculature (Supplementary Figures 3X,AA,DD).

### A Quintuple *shi/sty* Mutant Shows an Assortment of Aberrant Stamen Morphologies

**FIGURE 4:**
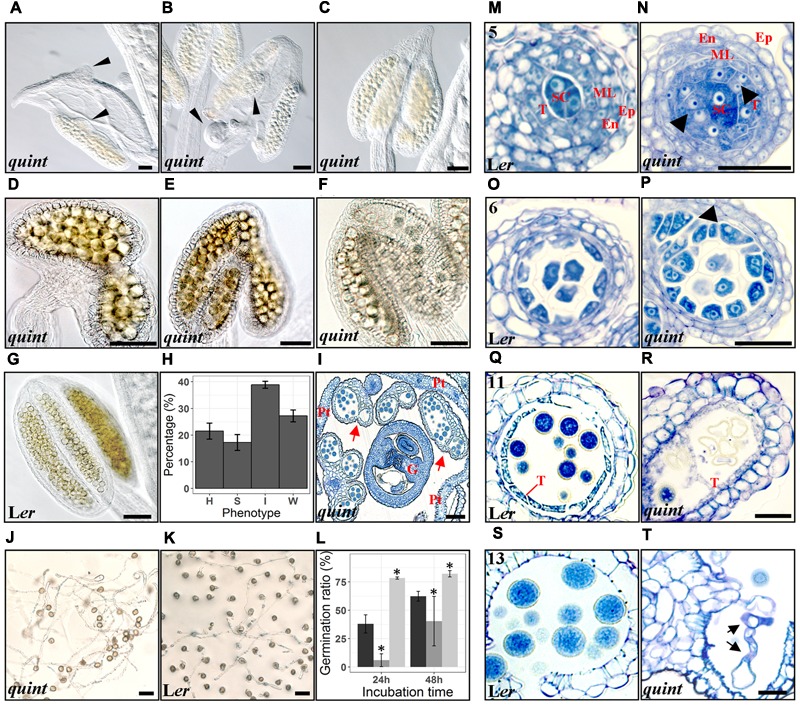
The quintuple *shi/sty* mutant shows a variety of defects in anther and pollen development. **(A–C)** Homeotic conversions of flower organs of the quintuple mutant, thin black arrowheads indicate ovule-like protrusions. **(D–F)** Strong, intermediate and wild type-like phenotypes, respectively, of mutant anthers. **(G)** A wild type L*er* anther. **(H)** shows a bar plot of the percentage of each phenotype in the quintuple mutant, with error bars representing the standard error of the mean of at least 30 different floral buds. H, Homeotic; S, Strong; I, Intermediate; W, Weak. **(I)** Toluidine Blue stained cross section of a quintuple floral bud. G, Gynoecium; Pt, Petal; red arrows indicate anthers with aberrant locules. **(J,K)** Germinating pollen from the quintuple mutant and wild type L*er* respectively, after 48 h of culture. **(L)** Bar plot of germination ratio percentages. Mean ± SD values of three independent experiments are represented by black (Col) dark gray (L*er*) and light gray (quintuple *shi/sty* mutant) bars. Asterisks indicate significant means according to Kruskal-Wallis test and α = 0.05. **(M–T)** Microsporangia cross-sections stained with Toluidine Blue at anther stage 5 **(M,N)**, 6 **(O,P)**, 11 **(Q,R)** and 13 **(S,T)**. Wild type L*er* sections are shown in the left panel with numbers indicating anther stages **(M,O,Q,S)** and quintuple sections at corresponding stages in the right panel **(N,P,R,T)**. Thick black arrowheads point to excessive tapetal cells. Black arrows point to germinating pollen tubes within the anther. SC, Sporogeneous Cells; T, Tapetum; ML, Middle layer; En, Endothecium; Ep, Epidermis. Bars **(A–L)** = 60 μm, Bars **(M–T)** = 20 μm.

As no single *shi*/*sty* mutant shows anther phenotypes (Kuusk et al., 2006; Kim et al., 2010), the distinct, but in several cases also overlapping expression pattern of the *SHI*/*STY* genes during anther development suggests that they act partially redundant. Thus, to further assess their role in stamen development, we used a quintuple line carrying insertion mutations in *STY1, STY2, SHI, LRP1* and *SRS5* (Kuusk et al., 2006). Previous work reports homeotic conversions of petals and stamens of this mutant (Kuusk et al., 2006), We found that 18–25% of the quintuple anthers show homeotic conversions including organs of mixed petal-stamen identity or anthers with gynoecial characters such as ovule-like protrusions from microsporangia or from carpeloid structures attached to underdeveloped filaments (**Figures 4A–C,H**). The remaining anthers were frequently affected in the locule formation, resulting in disproportionate locule sizes and reductions in locule number. We classified these defects as strong (14–20%) when most of the locules were missing or not easily recognizable, and intermediate (37–40%) if the anther had an arrow-shaped structure with some locular compartments (**Figures 4D,E,H,I**). In some cases the intermediate anthers carry lateral protrusions of microsporangia tissue or bifurcated vasculature. Anthers closely resembling wild type were classified as weak (25–30%) (**Figures 4F,H** compare to **Figure 4G**).

### Mutations in Multiple *SHI/STY* Genes Results in Multi-Layered Tapetum Proliferation and a Decrease in Pollen Viability

Histological analyses of quintuple mutant anthers suggest that *SHI*/*STY* genes affect tapetal proliferation and pollen viability, predominantly in the homeotic and strong classes. Already at stage 5 a deviating tapetal cell division pattern is apparent in the quintuple mutant and at stage 6–7 it is clear that the tapetum is partly multi-layered (compare **Figures 4M,O** and **Figures 4N,P**). At later stages, a fraction of anthers exhibit an aberrant over-proliferating tapetum filling the inner parts of the locule and seemingly empty collapsed microspores with irregular exine walls were occasionally observed (compare **Figures 4Q,R**). Since the tapetal cell division defects are clear already at stage 5, they are probably caused by events occurring very early (stages 3–4), when the *SHI*/*STY* genes are expressed throughout the stamen primordia.

*SHI*/*STY* genes are also expressed in the tapetum and the microspores at later developmental stages suggesting that they might affect additional processes during pollen development and stamen maturation. To assess pollen viability we carried out Alexander staining assays (Peterson et al., 2010). These revealed pollen viability close to 100% in wild type. However, in approximately 30% of the homeotic or strongly affected quintuple mutant anthers, the majority of the pollen stained in blue and was therefore not viable (Supplementary Figure 4). The non-viable pollen grains were usually smaller and/or more irregularly shaped compared to wild type (Supplementary Figure 4C). We also performed an *in vitro* germination assay (Li et al., 1999) to assess the germination capability of mature quintuple mutant pollen. At anthesis, pollen was harvested from the quintuple mutant and from two of the background wild-type ecotypes crossed into the quintuple mutant line, Columbia (Col) and *L. erecta* (L*er*). Of the pollen accessible for harvest, which excludes pollen from the most severely affected quintuple anthers, those of the quintuple mutant showed a higher germination rate (75–80%) compared to the two wild type ecotypes (60–65% in Col and 20–60% in L*er*) 48 h after incubation (**Figures 4J–L**). Precocious pollen germination was occasionally also observed inside anthers of the quintuple mutant (compare **Figures 4S,T**), suggesting that the SHI/STY proteins act in a pathway that induces or maintains pollen dormancy. The pollen tube length of the quintuple mutant pollen is similar to Col (22% versus 27% of the pollen tubes measure less than 200 μm, 14.5% versus 9.4% measure between 200 and 400 μm and 63.6% of both lines measure more than 400 μm), suggesting that loss of *SHI/STY* activity, especially *STY1* and *LRP1* that show activity in mature pollen (**Figure 3**), does not impede pollen tube elongation *in vitro*.

### The Quintuple *shi/sty* Mutant Undergoes Premature and Enhanced Endothecial Lignification and Tapetal PCD

**FIGURE 5:**
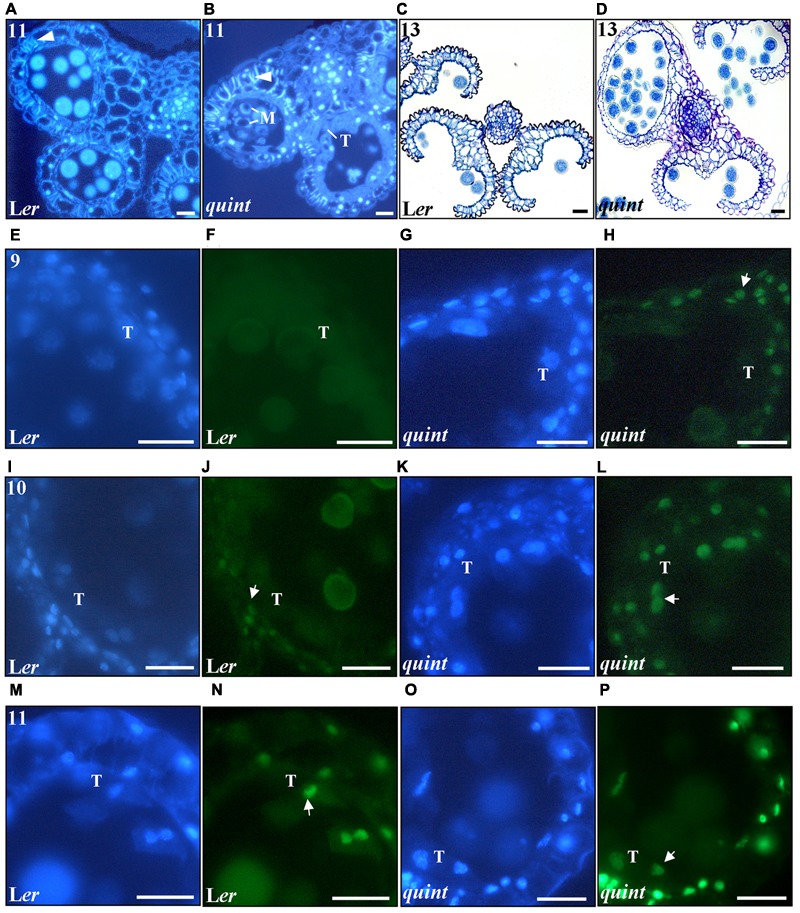
The multiple *shi/sty* mutant shows premature and enhanced endothecial lignification, premature DNA fragmentation in anther cells and impaired stomium opening. Cross-sections of DAPI-marked stage 11 anthers of **(A)** wild type L*er* and **(B)** the quintuple mutant. White arrowheads point to lignified thickenings. M, vacuolized non-viable microspores; T, aberrant tapetal cells. **(C,D)** Toluidine Blue stained cross-sections of stage 13 anthers of **(C)** wild type L*er* and **(D)** quintuple mutant depicting stomium opening. **(E–P)** Fluorescence images of **(E,F,I,J,M,N)** wild type L*er* and **(G,H,K,L,O,P)** quintuple mutant 8 μm cross sections showing DAPI (blue field) and TUNEL (green field) signals. **(E–H)** correspond to stage 9, **(I–L)** correspond to stage 10 and **(M–P)** correspond to stage 11. Numbers indicate anther stages and white arrows point to TUNEL emitting nuclei. T, Tapetal cell nucleus. Bars = 20 μm.

Lines carrying mutations in several of the *TIR*/*AFB* auxin receptor genes show alterations in pollen maturation and endothecium lignin deposition (Cecchetti et al., 2008). We carried out a comparative histological study to assess the timing of endothecial lignin deposition in the quintuple mutant using the process of pollen development as a stage reference. In Arabidopsis anthers, lignin deposition occur in the endothecium at stage 11, when the generative cell has entered mitosis and the pre-pollen is at late bi-cellular phase, long after the large vacuole has been split into smaller ones (Owen and Makaroff, 1994; Sanders et al., 1999; Borg et al., 2009). Our observations suggest that endothecial lignification occurs prematurely in the quintuple mutant, already when the pre-pollen is in an early bi-cellular stage and some of the microspores still contain vacuoles at stage 10 (Supplementary Figures 5B,F). Moreover, endothecium lignification in the quintuple anthers is more abundant compared to wild type and seems to be intensified by the severity of the quintuple anther phenotype (**Figures 5A,B**). Several *SHI*/*STY* genes are expressed in the endothecium at stages before lignification is visible, suggesting that their action may be required to repress premature lignification.

As PCD is involved in the degeneration process of the endothecial and tapetal cells (Vizcay-Barrena and Wilson, 2006; Zhu et al., 2011; Fernández Gómez et al., 2015), we assessed the timing and degree of nuclear DNA degradation using transferase mediated dUTP nick-end labeling (TUNEL) in wild type and the quintuple mutant (**Figures 5E–P**). In wild type, the first TUNEL signals were detected at stage 10, and were observed only in the endothecium nuclei (**Figures 5I,J**) and in the vascular bundle (data not shown). Concomitant with the lignin deposition at stage 11, the TUNEL signals intensified in the endothecial nuclei and were observed in the nuclei of the epidermis and tapetal cells (**Figures 5M,N**). In contrast, TUNEL signaling was observed prematurely in the quintuple mutant. Already at stage 9, endothecial and vasculature signals were observed (**Figures 5G,H**). At stage 10, TUNEL signal increased in these cells and extended to the epidermis and tapetal nuclei (**Figures 5K,L**). At stage 11 intensified TUNEL signals were detected practically in the whole anther, with the strongest signal in the cell layers surrounding the locules (**Figures 5O,P**). These results show that the *SHI/STY* genes not only repress premature lignification, but also premature and ectopic PCD in the anther outer cell layers.

### Loss of *SHI/STY* Activity Affects Stomium Differentiation Leading to Impaired Anther Opening and Pollen Release Capability

At late stages of Arabidopsis anther development (stage 12) the septum lyses and the stomium, a specialized group of cells originating from the epidermis, opens through a process of cell death and separation. This allows the release of mature pollen (for a review see Wilson et al., 2011). In quintuple mutant anthers that lack a locule or show deviating locule sizes, differentiation of stomium cells is defective or lacking completely in some parts. As a consequence the pollen is not released but kept inside the locules (**Figures 5C,D**).

### Auxin Sensing Occurs Throughout Anther Development and Overlaps with *SHI*/*STY* Expression Profiles

Spatiotemporal analysis of auxin responses in anthers using *DR5:GUS* (Ulmasov et al., 1997) and *DR5:GFP* (Blilou et al., 2005) did not reveal any reporter signals at anther stages 1–7 (anthers of floral stages 5–9), except for in most apical cells at stage 7 (Cecchetti et al., 2008). By contrast, both *SHI*/*STY* and *YUC* genes (Cheng et al., 2006) are expressed already at early stages of anther development suggesting that auxin mediated information may be important also before stage 8. This prompted us to use auxin-sensing reporters suggested to be more sensitive. We selected the *DR5rev:GFP* reporter (Friml et al., 2003), as well as the ARF-independent *R2D2* auxin sensor system. The *R2D2* system is based on a single construct expressing an auxin sensitive Aux/IAA degradation domain (DII) linked to the fluorophore Venus, DII-n3x-Venus (green), and an auxin-resistant mutated version linked to the fluorophore Tomato, mDII-ndtTomato (magenta), both driven by the *RPS5S* promoter (Liao et al., 2015). Auxin sensing readouts from auxin-mediated Aux/IAA depletion can thus be measured as the relative cellular accumulation of Venus/Tomato signals, the lower the ratio, the higher auxin sensing.

**FIGURE 6:**
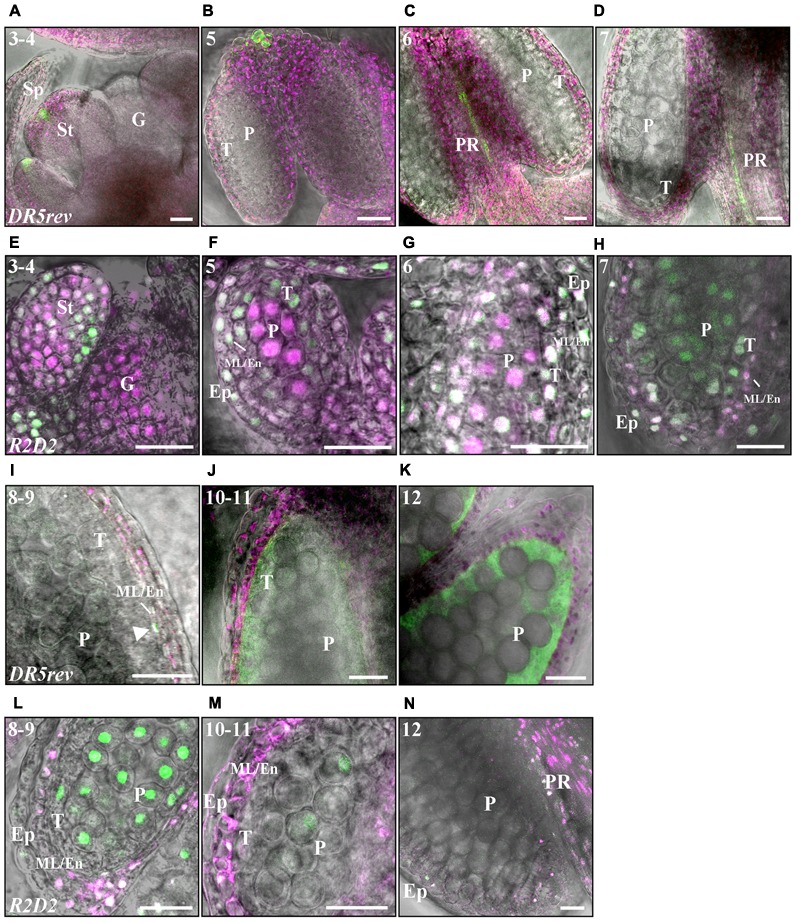
Fluorescence-imaging of *DR5rev:GFP* and *R2D2* reveals auxin sensing in early and late anther development. **(A–D,I–K)** *DR5rev:GFP* expression in merged GFP fluorescence (green), chloroplast autofluorescence magenta) and DIC pictures. **(E–H,L–N)** *R2D2* activity in Venus/Tomato (green/magenta) and DIC composite pictures. Numbers indicate anther stages. **(A,E)** Stage 3-4, **(B,F)** Stage 5, **(C,G)** Stage 6. **(D,H)** Stage 7. **(I,L)** Stage 8–9. **(J,M)** Stage 10–11. **(K,N)** Stage 12. Letters: Sp, Sepal; St; Stamen; G, Gynoecium; T, Tapetum; ML/En, Middle layer/Endothecium; Ep, Epidermis; P, Pre-pollen; PR, Procambium. The white arrowhead in **(L)** points to a ML cell with GFP signal. Bars = 20 μm.

Indeed, the *R2D2* construct reveals that auxin-mediated DII depletion occurs in the locules at very early developmental stages. A low DII-Venus/mDII-Tomato fluorescence ratio in all internal cells of stage 3–4 anther primordia (**Figure 6E**) indicates that auxin is sensed in these tissues. In addition, *DR5rev:GFP* (**Figures 6A,B**) but not *DR5:GUS* or *DR5:GFP* (Aloni et al., 2006; Cecchetti et al., 2008), is active in epidermal tip cells already at these early stages where it is maintained throughout anther development (Supplementary Figure 6). This early auxin sensing overlaps with *STY1, STY2, SHI, SRS5* and *SRS7* expression (**Figure 2** and Supplementary Figure 3) as well as with *YUC1* and *YUC4* activity (Cheng et al., 2006; Ståldal et al., 2012).

Between stages 3 and 5 DII depletion becomes successively reduced in the differentiating cells surrounding the inner sporogenous cells while it remains high in the PMCs (**Figure 6F**), suggesting that auxin dynamics may be important for outer cell layer differentiation that takes place at this stage as well as for events occurring in the PMCs. A low DII-Venus/mDII-Tomato fluorescence ratio remains at stage 6 in the PMCs and the tapetum (**Figure 6G** and Supplementary Figures 6J,K) as well as in the procambium, where it overlaps with *DR5rev:GFP* activity (**Figure 6C**) and coincides with *YUC2* and *YUC6* expression (Cecchetti et al., 2008).

After meiosis, which results in tetrad formation (stage 7) (**Figures 6D,H**), the auxin-mediated DII depletion is drastically reduced in the gametophytic cells (**Figure 6H**). In contrast, GUS staining in *DR5:GUS* gametophytic cells were not observed until stage 8, when microspores are released and GUS staining was detected in microspores until stage 11 (Cecchetti et al., 2008, 2017). While auxin-mediated DII-depletion reappears in the ML and endothecial cells at stage 7, it is reduced in tapetal cells and remains low in the epidermis (**Figure 6H** and Supplementary Figures 6L,M). Low DII-Venus/mDII-Tomato fluorescence ratio is maintained in the ML and endotethium throughout stages 8–11 (**Figures 6L,M**). In accordance, at stage 8, when the first internal expression of *DR5* reporters could be observed, a transient peak of *DR5:GUS* (Cecchetti et al., 2017) and *DR5rev:GFP* activity (arrows in **Figure 6I** and Supplementary Figures 6B,C) could also be detected in the ML and endothecial cells. This coincides with high *SRS7* activity in the ML and endothecium. While the *DR5* reporters are active in the tapetum around stages 9–11 (**Figure 6J**) (Aloni et al., 2006; Cecchetti et al., 2008), overlapping with *STY1, STY2, LRP1* and *SRS7* activity (**Figure 3**), only very low *R2D2* fluorescent signals can be observed in the tapetal cells (**Figure 6M**). This suggests that the *RPS5A* promoter activity is very low during tapetal PCD. Remaining *DR5rev:GFP* mediated GFP signals was observed in tapetal cell remnants and exine walls of mature pollen at late stage 11 to early stage 12 (**Figures 6J,K** and Supplementary Figure 6D), coinciding with remains of GUS-staining in the *LRP1, STY1* and *STY2* reporter lines, presumably representing material deposited from the tapetum.

At stage 12, DII depletion can only be detected in the apical part of the filament overlapping with high *DR5rev:GFP* and *SHI*/*STY* activity (**Figure 6N**). However, at these late stages, the *RPS5A* promoter is almost inactive in some cell layers as neither DII-Venus nor mDII-Tomato can be detected (**Figure 6N** and Supplementary Figures 6N,O), making the *R2D2* sensor system less informative at these stages. At stage 13, high *DR5rev:GFP* expression is detected in the vasculature (Supplementary Figure 6E) overlapping with *LRP1, SRS6* and *SRS7* (Supplementary Figure 3).

In summary, the map of auxin-mediated readout obtained in this study using the *R2D2* sensor both overlaps and complements *DR5* activity domains. It suggests that an auxin distribution gradient is established that determines anther cell type differentiation at stage 5, resulting in high auxin sensing at the PMCs. This suggests an important role of auxin during the meiosis and tetrad formation. At stage 6, when meiosis occurs, also the tapetum shows auxin-mediated readout. After meiosis, a transition toward a reduced auxin sensing occurs in the gametophyte, whereas high auxin sensing is transferred to ML and endothecium preparing these tissues for the impending dehiscence processes.

### The Activity of the STY1 Targets *EOD3, PAO5* and *PGL1* Overlap with *SHI/STY* Expression

**FIGURE 7:**
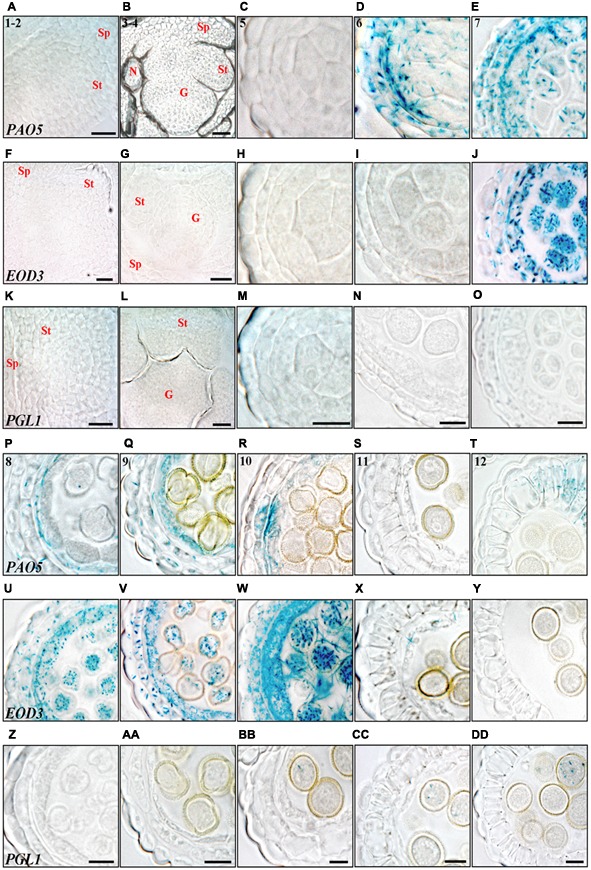
Histochemical analysis of promoter activity of STY1 targets through anther development. **(A–E,P–T)** *PAO5pro:GUS*, **(F–J,U–Y)** *EOD3pro:GUS* and **(K–O,Z–DD)** *PGL1pro:GUS* activity visualized as blue staining in cross-sections of floral buds (stages 1–2 and 3–4) and microsporangia (stage 5–12). Numbers in *PAO5pro:GUS* microsporangia cross-sections indicate anther stages. **(A,F,K)** Stage 1–2. **(B,G,L)** Stage 3–4. **(C,H,M)** Stage 5. **(D,I,N)** Stage 6. **(E,J,O)** Stage 7. **(P,U,Z)** Stage 8. **(Q,V,AA)** Stage 9. **(R,W,BB)** Stage 10. **(S,X,CC)** Stage 11. **(T,Y,DD)** Stage 12. Letters indicate floral parts or their primordial equivalents, St, Stamen; G, Gynoecium; Sp, Sepal; N, Nectary. Bars at stages 1–2 and 3–4 = 20 μm. Bars at stages 5–12 = 10 μm.

Of the putative direct STY1 targets (Ståldal et al., 2012), *PGL1* showed the strongest response to STY1 overexpression and *PAO5* had already been indicated to be important during anther development (Fincato et al., 2012). However, expressional analysis of the STY1 direct targets *EOD3, PAO5* and *PGL1* suggest that these genes are not involved in stamen emergence or early specification of internal cell layers as they are only active after stage 5 (**Figure 7**).

*PAO5* is strongly activated at stage 6 where it overlaps with a peak of *SRS7* and *STY2* expression in epidermis, endothecium, ML and the PMCs (**Figures 7D, 2I,X**). From stage 8–9, *PAO5* is mainly active in the tapetum, overlapping with several *SHI/STY* genes (**Figures 7P,Q, 3B,F,G,K,L,P,Q** and Supplementary Figures 7C–E), suggesting that PAO5 is important for regulating the timing of tapetal cell fate. PAO5 has been shown to catalyse the conversion of T-Spm or Spm to Spd and changes in PAs homeostasis can affect PCD processes (Kim et al., 2014; Moschou and Roubelakis-Angelakis, 2014). At stage 12 *PAO5* is expressed in lignified endothecial cells in the abaxial part of the anther but not in pollen (**Figure 7T** and Supplementary Figures 7G,H). This suggests that PAO5 may be important for changing the mechanical properties of the abaxial endothecium to allow stomium opening. As *PAO5* expression overlaps with *SRS7* in endothecial cells (**Figure 3Y**), SHI/STY family members may be important regulators of *PAO5* in this tissue. *PAO5* is also active in the apical part of the anther filament, where it overlaps with *LRP1, SRS6* and *SRS7* from stage 5 until pollen release (Supplementary Figures 3, 7A–F). This suggests that PAO5 may play a role in anther filament vasculature development, supported by the recent implication of PAO5 in the auxin and cytokinin interplay required for proper xylem differentiation (Alabdallah et al., 2017).

*EOD3* becomes active slightly later compared to *PAO5* and is highly active in all cell layers, except the epidermis, from stage 7 to stage 10, strongly overlapping with *STY2* expression (**Figures 7J,U–W** and Supplementary Figures 7I–M). At stage 10 a transient peak of *EOD3* expression occurs in the microspores and the surrounding thick tapetum and ML (**Figure 7W** and Supplementary Figure 7M). It has been suggested that *EOD3* promotes ovule integument cell expansion and proliferation affecting seed size (Fang et al., 2012). Similarly, *EOD3* could play a role in the processes of tapetal expansion and vacuolization allowing the stockpiling of lipidic compounds to be transferred to the microspores (Piffanelli et al., 1998). Accordingly, little or no *EOD3* activity can be observed in the microspores at stage 11 or later when tapetal degradation is completed (Supplementary Figures 7N–P).

*PGL1* is active in the germ cell line where its expression overlaps mainly with that of *STY1, LRP1* and *SRS7*. *PGL1* is activated when the microspores have reached the binuclear stage, and peaks in the trinuclear stage after which it remains active during pollen germination (**Figures 7BB–DD** and Supplementary Figures 7Q–T). The activity in pollen germination may give some support to PGLs suggested role as a cell-wall protein modifier (da Costa-Nunes and Grossniklaus, 2003; Pina et al., 2005). In addition, *PGL1* becomes active at the anther tip at stage 12, where it overlaps with *STY1, STY2, SHI, SRS5* and *SRS7* and its expression increases at stages 13–14 (Supplementary Figures 3, 7Q,R).

## Discussion

In this work we have carried out a comprehensive study of the expression domains of different *SHI*/*STY* genes and three of their potential direct targets during anther development. We have also characterized the phenotype of a quintuple *shi/sty* mutant to shed light on the role of this gene family during the development of the male reproductive organ. Included in our studies is a first approach to obtain a map of ARF-independent auxin sensing complementing the previous knowledge acquired using *DR5*-based strategies in anthers.

### The ARF-Independent *R2D2* Auxin Sensing Construct Reveal Early Auxin Activities during Anther Development

Hitherto, *DR5* promoter-based auxin signaling reporters has been used to setup anther auxin-sensing maps suggesting that auxin responses are negligible during the early stages of anther development (stages 1–7) (Aloni et al., 2006; Feng et al., 2006; Cecchetti et al., 2008, 2017). Using the *R2D2* auxin sensor we could show that auxin-mediated Aux/IAA depletion also occurs at stages 1–7. This prompted us to map auxin-readout throughout anther development to further our understanding of the role of auxin at all developmental stages.

First of all, using the *DR5rev:GFP* and *R2D2* auxin-readout reporters, we could show that these reveal auxin sensing in exactly the same cell types as has been suggested by the *DR5:GUS* expression pattern at stage 8, the earliest stage when *DR5:GUS* is detectable. This clearly indicates that the *R2D2* is indeed a useful auxin sensor in anthers. At stage 8, when the microspores are released, *DR5:GUS* (Cecchetti et al., 2008, 2017), *DR5rev:GFP* and *R2D2* show responses indicating auxin signaling activities in the endothecium and ML. At this stage, *ABCB1* and *ABCB19*, encoding auxin efflux carriers, are active only in the tapetum, microspores and procambium (Cecchetti et al., 2008, 2015). Using the auxin transport inhibitor NPA, Cecchetti et al. (2017) could show that active auxin transport is required to create the auxin response peak in the ML, and that without active transport, the peak occurs in the tapetum layer instead suggesting that ABCB-mediated transport of auxin from the tapetum provides the ML and endothecium with auxin.

We could show that already at stage 7, when meiosis is just completed, auxin-mediated readouts detected by *R2D2* peaks in ML and endothecial cells. *YUC2* is also active in these cells while the *ABCB* genes are expressed only in the tetrads, tapetum and procambium (Cecchetti et al., 2008, 2015), suggesting that auxin transport from tapetum to, and auxin sensing in, ML and endothecium occurs already at this stage. In contrast, at an earlier stage, when the PMCs enter meiosis (stage 6), we could detect strong auxin sensing in the PMCs and procambium, and a weaker sensing in the tapetum. At this stage, auxin-mediated readout strongly overlaps with *ABCB1, ABCB19, YUC2* and *YUC6* expression (Cecchetti et al., 2008, 2015), suggesting that auxin is both produced and sensed in as well as exported from PMCs, tapetum and procambium. This indicates that auxin homeostasis and signaling may be important for PMC meiosis as well as for tapetum and procambium development. Indeed, *abcb1abcb19* anthers show asynchronous meiotic progression and altered tapetum proliferation (Cecchetti et al., 2015). Earlier, at stage 5, auxin sensing is mainly detected in the PMCs. Furthermore, the observed DII depletion suggests that an auxin maximum is established already in the emerging stamen primordia, at the tip as well as in the procambium. In addition, auxin is sensed in all cell-layers of stage 3–4 anther primordia, overlapping with *YUC4* activity and weak expression of *ABCB19* (Cheng et al., 2006; Cecchetti et al., 2015). This may suggest that auxin gradients are important for cell type specification, similarly to what has been observed around the QC in the root (Liao et al., 2015).

Interestingly, the observed overlap between auxin responses detected by *DR5:GUS* and the *R2D2* auxin readout system at stage 8 is only transient. Auxin-mediated DII-depletion was maintained in the ML and endothecium from stages 7 through 11, while at stage 9, when microspores carry an exine wall, *DR5:GUS* is no longer detected in the ML, is only weakly expressed in the endothecium, while it is strongly active in the tapetum, microspores and procambium (Cecchetti et al., 2017). This discrepancy could be based on the activity of specific ARF proteins, required for transcriptional activation of the *DR5*-promoter, while the *R2D2* sensor is ARF independent. At later stages (stage 10 and 11) when tapetum degeneration is initiated, *DR5*-mediated GUS staining remains in the tapetum and procambium (Cecchetti et al., 2017). As the *PRS5A* promoter driving the expression of DII-Venus and mDII-Tomato is low in degenerating tapetum cells, it is likely that *DR5* is also inactive and that the detected GUS-staining represents remains from stage 9 *DR5* activity.

### Mutations in *SHI*/*STY* Genes Results in Defective Anther Development

The expression analysis of GUS-reporter constructs of six of the nine highly redundant Arabidopsis *SHI*/*STY* genes reveal that all six are expressed during anther development and that their expression domains to some degree are overlapping. Using the *sty1-1 sty2-1 shi-3 lrp1 srs5-1* quintuple mutant (Kuusk et al., 2006) we could characterize some of the developmental phases where *SHI*/*STY* gene activity is required for developmental decisions. First of all, *SHI*/*STY* expression in very early anther primordia is needed for proper specification of anther identity, anther locule number and locule sizes. So far auxin has been implicated in organ initiation and growth but not in organ identity specification (Tobena-Santamaria et al., 2002; Cheng et al., 2006; Krizek, 2011), therefore it is likely that other downstream targets of the SHI/STY transcription factors are mediating these SHI/STY functions.

We could also detect deviating tapetal cell proliferation in the quintuple mutant, already at stage 5, resulting in multiple layers of tapetum cells at stages 6–7. As mentioned above, strong auxin sensing, as well as *YUC* and *ABCB* activities takes place in the tapetum at stage 6, and weak activities could also be observed at stage 5. The *abcb1 abcb19* double mutants show a similar over-proliferation of tapetum cells (Cecchetti et al., 2015) observed at stages 7–8 suggesting that auxin efflux from the tapetum layer at earlier stages may be important to prevent periclinal cell divisions in the tapetal cell file. The phenotypic similarity between the quintuple mutant and the *abcb1 abcb19* double mutant suggest that the SHI/STY proteins may affect auxin homeostasis in tapetal cells at these stages.

In addition, at later stages the *SHI*/*STY* genes appear important to synchronize pollen maturation with endothecial lignification and tapetum degeneration. When using the developmental phase of the gametophytic cells as a stage-specifying marker, lignin deposition in endothecial cells and nuclear DNA degradation in endothecial and tapetal cells occurs prematurely in the quintuple mutant anthers, at stage 10, when *SHI*/*STY* genes are still strongly expressed in the corresponding wild type cells. This is in agreement with the observed delay in tapetum degeneration and anther dehiscence in *SHI*/*STY* over-expressing lines (Kim et al., 2010). Interestingly, endothecial lignification was observed prematurely (stage 10) in relation to gametophytic cell progression also in mutants carrying mutations in the four genes encoding TIR/AFB auxin receptors (Cecchetti et al., 2008), suggesting that auxin signaling is required to prevent precocious lignification. Indeed, using *R2D2* we could observe that auxin is sensed in the wild type endothecium up until stage 10 and early 11, but not thereafter.

However, a corresponding premature onset of PCD in endothecial and tapetal cells detected in the quintuple mutant could not be observed in the *tir1 afb1 afb2 afb3* quadruple mutant. In contrast, the *tir1 afb1 afb2 afb3* gametophytic cells were more advanced in development compared to wild type when tapetal cell degeneration was initiated (Cecchetti et al., 2008). This may suggest that auxin signaling is inducing PCD initiation or progression in tapetal cells.

In the most strongly affected quintuple anthers, a large proportion of miss-shaped and non-viable pollen is formed. As *PGL1* is expressed in the microspores, this SHI/STY target may be important for providing the correct cell wall properties required for pollen morphogenesis, although *pgl1* single mutants could not verify this. A small fraction of aberrant non-viable pollen was also found in the *abcb1 abcb19* mutant (Cecchetti et al., 2015) suggesting that correct auxin homeostasis is important for pollen viability. Still, viable pollen of quintuple *shi/sty* mutants, *abcb1 abcb19* mutants, and of *TIR*/*AFB* quadruple mutants all show premature pollen germination (Cecchetti et al., 2008, 2015), implicating a clear role of auxin in inducing or maintaining pollen dormancy, thereby preventing precocious pollen germination.

## Conclusion

The work presented here shows that the *SHI*/*STY* genes play important roles during anther development. It reveals that *SHI*/*STY* genes play key roles in organ identity and cell type establishment at early stages that are essential for pollen development. It also shows that *SHI*/*STY* genes are important in controlling the timing of some aspects of anther development that are also regulated by auxin (such as repression of periclinal tapetum division, endothecium lignification and pollen germination) or through other pathways not connected to auxin action (such as pollen morphogenesis and repression of tapetal and endothecial PCD). Although the roles of the SHI/STY targets *EOD3, PAO5* and *PGL1* in anther development are unknown due to lack of single mutant phenotypes, their expression domains clearly suggest that they contribute to some of the SHI/STY functions in anther development. Our work also contributes to expand the current knowledge on the spatiotemporal activity of auxin sensing during anther development and suggests that auxin may contribute to a number of developmental decisions, from early anther primordial development to anther dehiscence.

## Author Contributions

LE designed most of the experiments except for the *PGL1* reporter constructs, which was designed by IC, and discussed these with KL and ES. LE performed most of the experiments, except the *PGL1* construct building and transformation. LE analyzed the data and discussed the results with KL and ES. LE wrote the manuscript. KL and ES commented on the manuscript.

## Conflict of Interest Statement

The authors declare that the research was conducted in the absence of any commercial or financial relationships that could be construed as a potential conflict of interest.
