# Supplementary material for: SHI/STY Genes Affect Pre- and Post-meiotic Anther Processes in Auxin Sensing Domains in Arabidopsis
Source: Front Plant Sci. 2018 Feb 14;9:150. doi: 10.3389/fpls.2018.00150 (PMC5817092; doi:10.3389/fpls.2018.00150)
Supplement: Supplementary file 1 [file Data_Sheet_1.docx]

Supplementary Material

*SHI*/*STY* genes affect pre- and post-meiotic anther processes in auxin sensing domains in Arabidopsis

**Leandro Hueso Estornell**, **Katarina Landberg**, **Izabela Cierlik**, **Eva Sundberg***

*** Correspondence:** Eva Sundberg: [eva.sundberg@slu.se](mailto:eva.sundberg@slu.se)

#
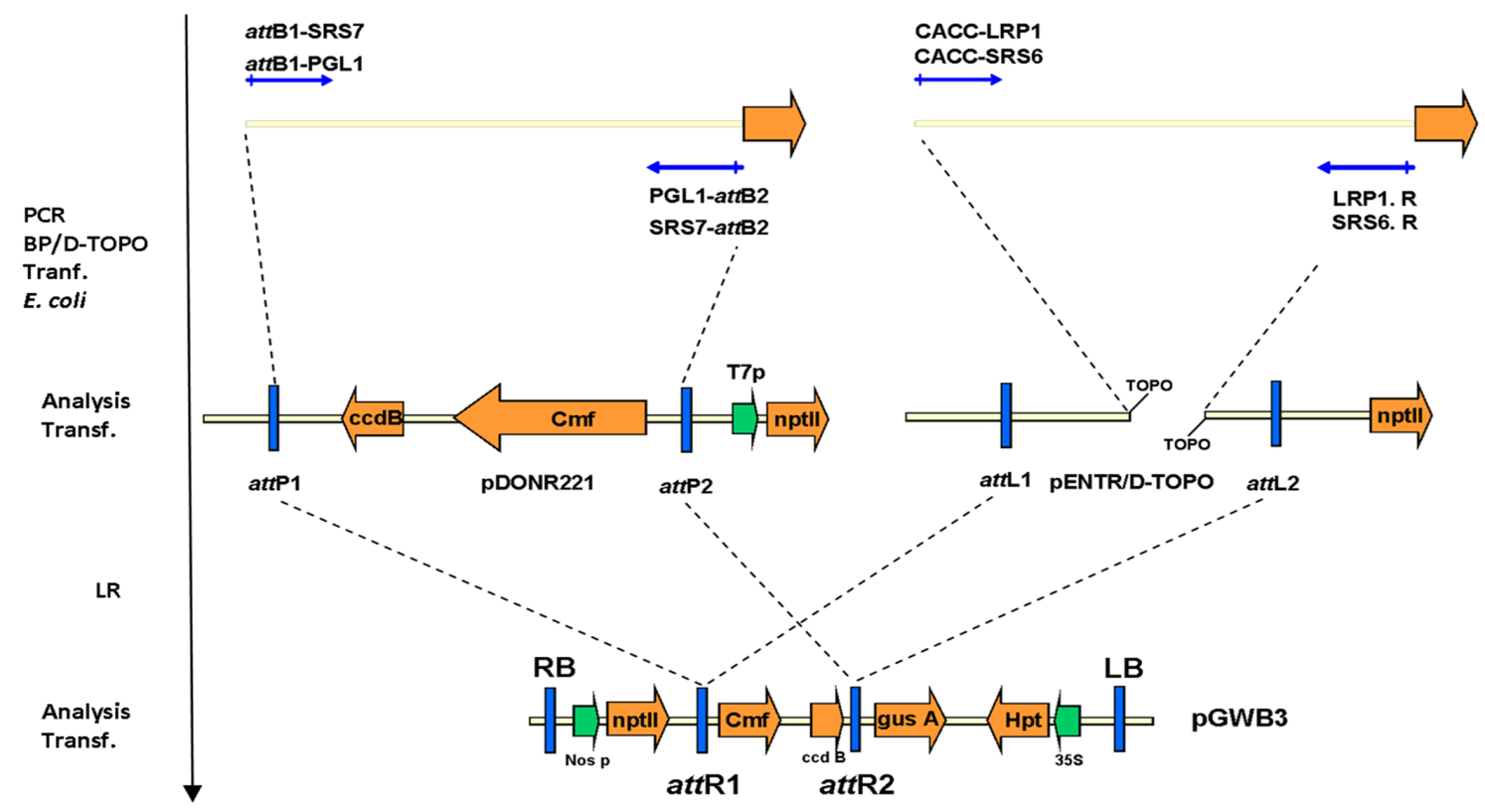
Supplementary Figures and Tables

## Supplementary Figures

**Supplementary Figure 1.** **Gateway cloning scheme**. For *PGL1* and *SRS7* promoter sequences long oligonucleotides with *att*B flanking sequences were designed to follow a BP recombination strategy, whereas a directional TOPO-Cloning was used in the case of *LRP1* and *SRS6* promoter sequences. A final LR recombination reaction allowed the fragments to be cloned into pGWB3. On the left side a schematic representation of the cloning steps ordered chronologically is provided.

**
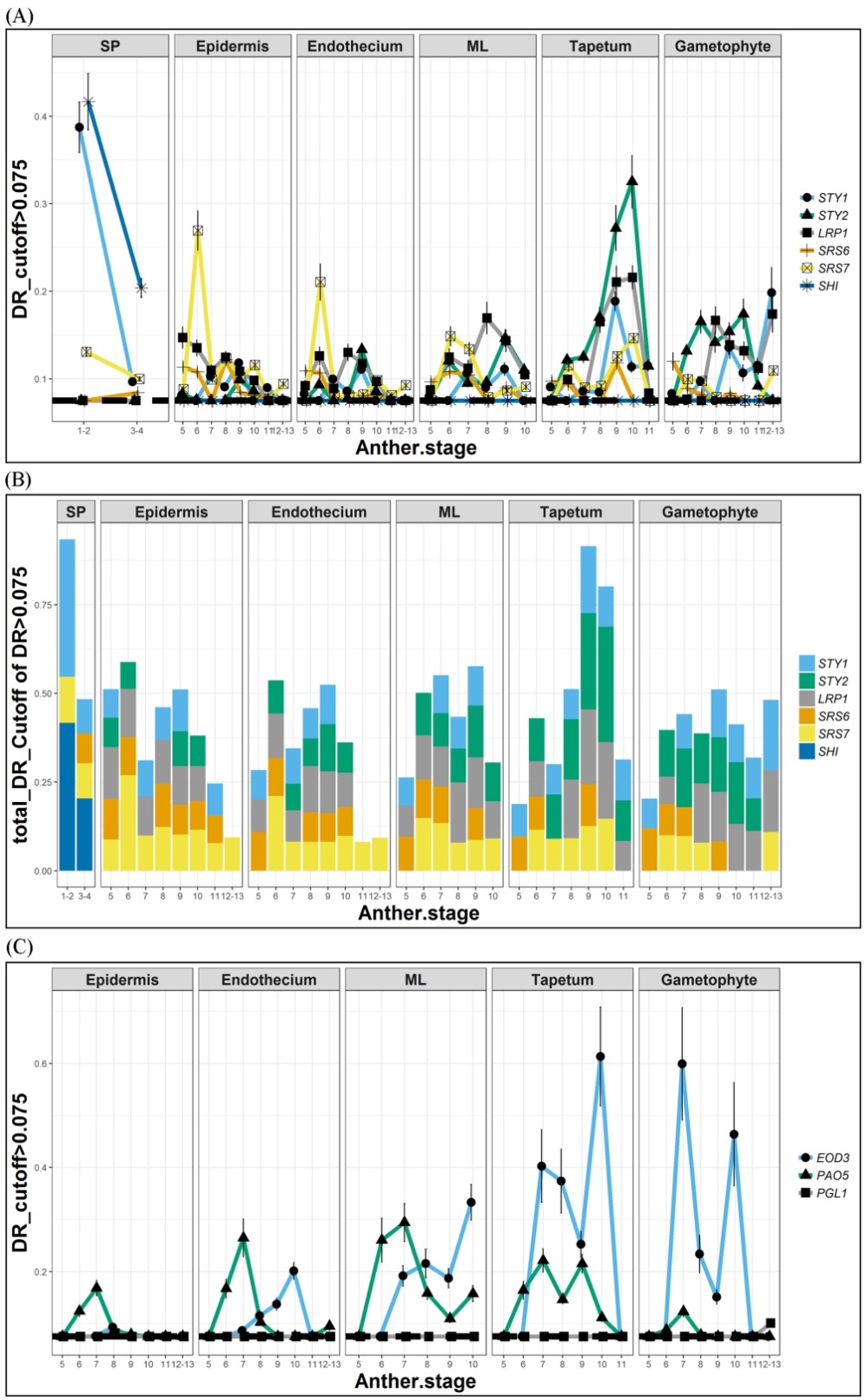
Supplementary Figure 2. Estimated promoter activity profiles throughout stamen development.** GUS intensity estimates based on the staining density in the red channel (D_R_) quantification (Pozhvanov and Medvedev, 2008) are represented by lines and bars with distinctive colors as indicated. Error bars in A and C represent the error associated to D_R_ values (m_DR_) calculated according to Pozhvanov and Medvedev, 2008. A baseline of DR=0.075 is represented as a black dashed line, values equal or below this value were regarded as zero. **(A-B)** GUS staining intensity profiles for *STY1pro:GUS*, *STY2pro:GUS*, *LRP1pro:GUS*, *SRS6pro:GUS*, *SRS7pro:GUS* and *SHIpro:GUS*. In **(B)** a stacked bar plot representing the DR values total sum is shown **(C).** GUS staining intensity profiles of *EOD3pro:GUS*, *PAO5pro:GUS* and *PGL1pro:GUS*. Samples with DR>0.075 in **(A)** are represented in **(B)**. SP: Stamen primordia, ML: Middle layer.

**
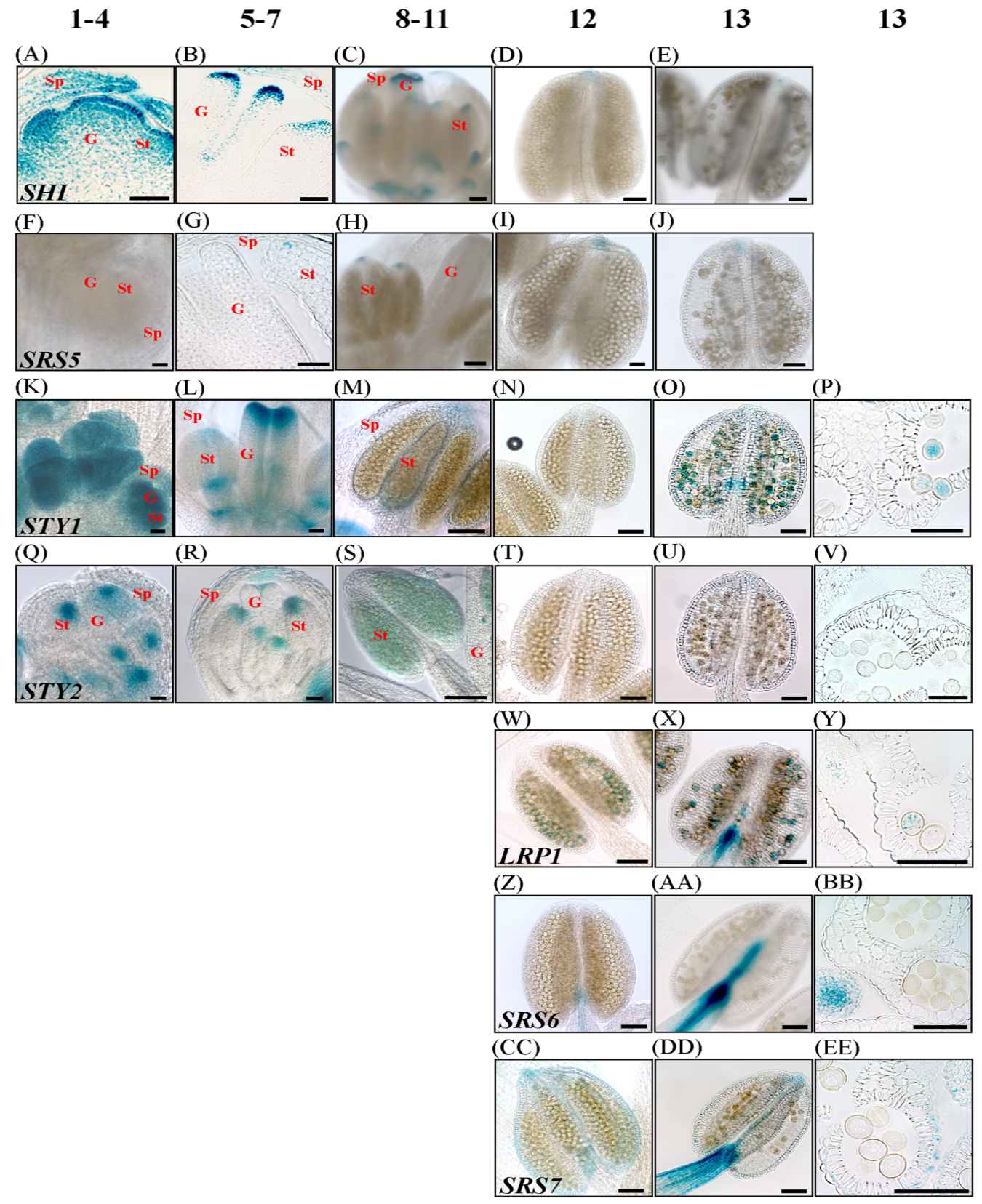
Supplementary Figure 3. Histochemical analysis of *SHIpro:GUS*, *SRS5pro:GUS*, *STY1pro:GUS*, *STY2pro:GUS*, *LRP1pro:GUS*, *SRS6pro:GUS* and *SRS7pro:GUS* promoter activities during stamen development.** Numbers above the figures represent anther stages. (**A-E**) *SHIpro:GUS.* (**F-J**) *SRS5pro:GUS*. (**K-P**) *STY1pro:GUS*. (**Q-V**) *STY2pro:GUS*. (**W-Y**) *LPR1pro:GUS*. (**Z-BB**) *SRS6pro:GUS*. (**CC-EE**) *SRS7pro:GUS*. (**A,B,G,P,V,Y,BB,EE**) are cross sections, while the rest of the images show whole mount floral buds or stamens. Sp: Sepal, St: Anther or stamen primordia, G: Gynoecium or gynoecium primordia. Bars in (**A,B,F,G,K,L,Q,R**) = 20 μm. Bars in the remaining images = 60 μm.

**
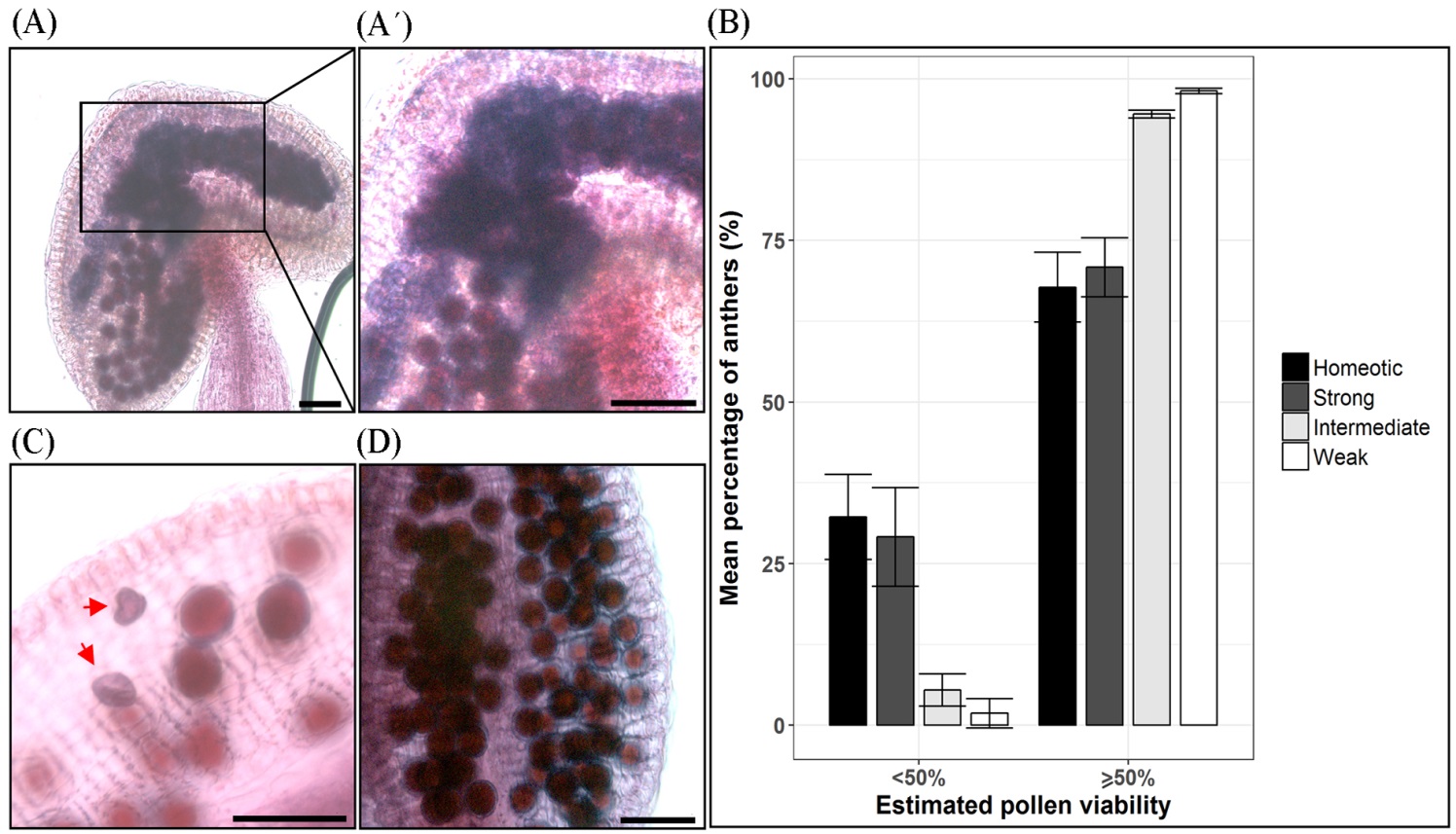
Supplementary Figure 4. Pollen viability is reduced in the *shi/sty* quintuple mutant.** (**A,C,D)** Stage 12 anthers stained with Alexander staining. (**A)** A strong quintuple mutant anther. (**A'**) show a higher magnification of the boxed area in (**A**). (**C)** Red arrows points to non-viable pollen in a quintuple mutant anther. (**D**) Wild type anther. (**B)** A plot of the mean of percentage of anthers according to the estimated pollen viability for each anther phenotype, bars representing the mean of the percentage of anthers ± se of three independent experiments. Bars = 50 μm.

**
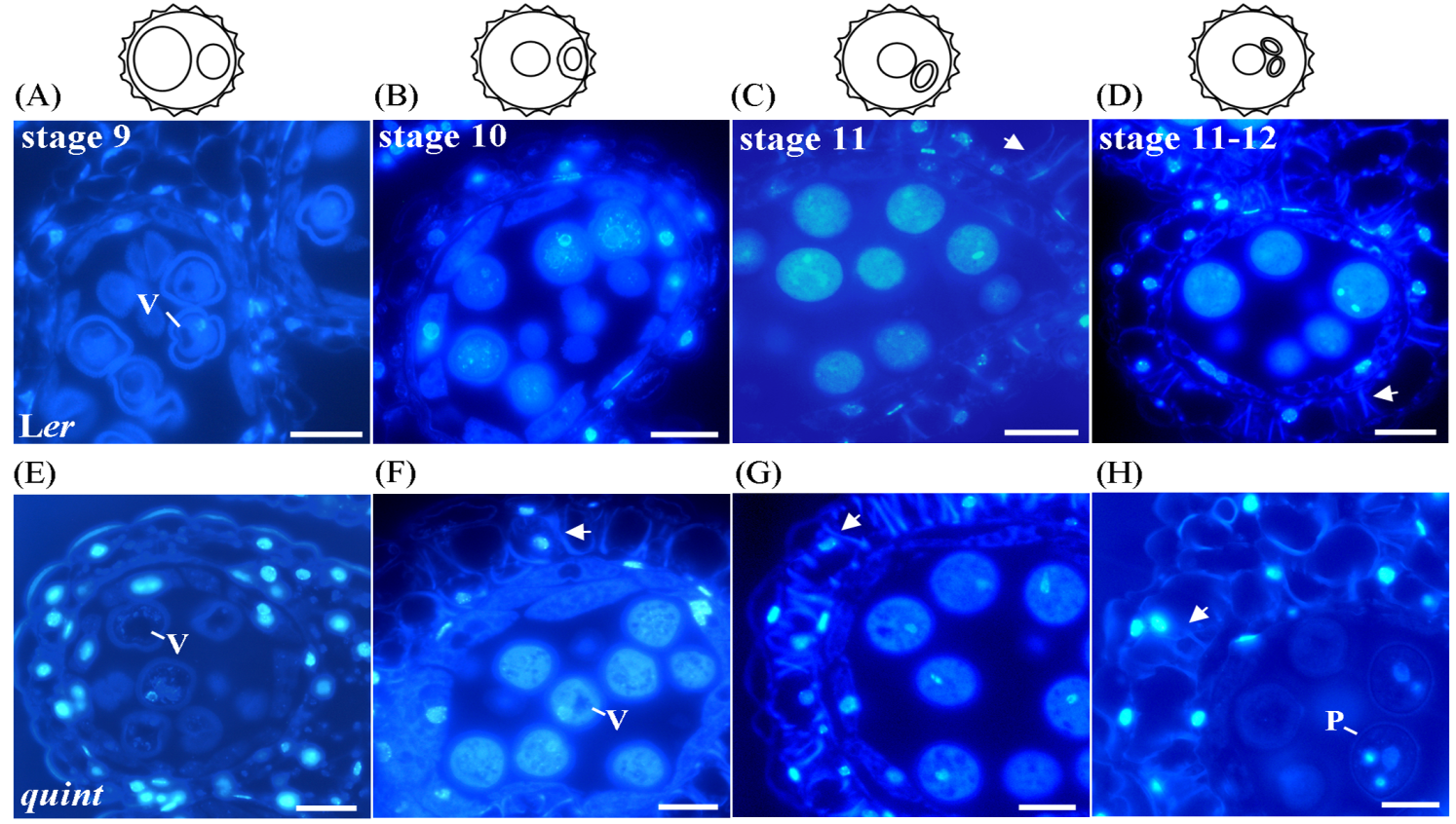
Supplementary Figure 5. The *shi*/*sty* quintuple mutant shows advanced endothecial lignification relative to pollen stages.** DAPI-stained 3 μm thick cross-sections from stage 9 to late stage 11 anthers were analyzed by fluorescence microscopy. (**A-D**) correspond to L*er* and (**E-H**) to the quintuple mutant. White arrows point to endothecial thickenings. V: Microspore vacuole, P: Trinuclear pollen. Pollen stages and drawings are based on Borg *et al*., 2009 and show a polarized microspore at stage 9, an early bicellular microspore at stage 10, a late bicellular microspore at stage 11 and a mature pollen at stage 11-12. Bars = 20 μm.

**
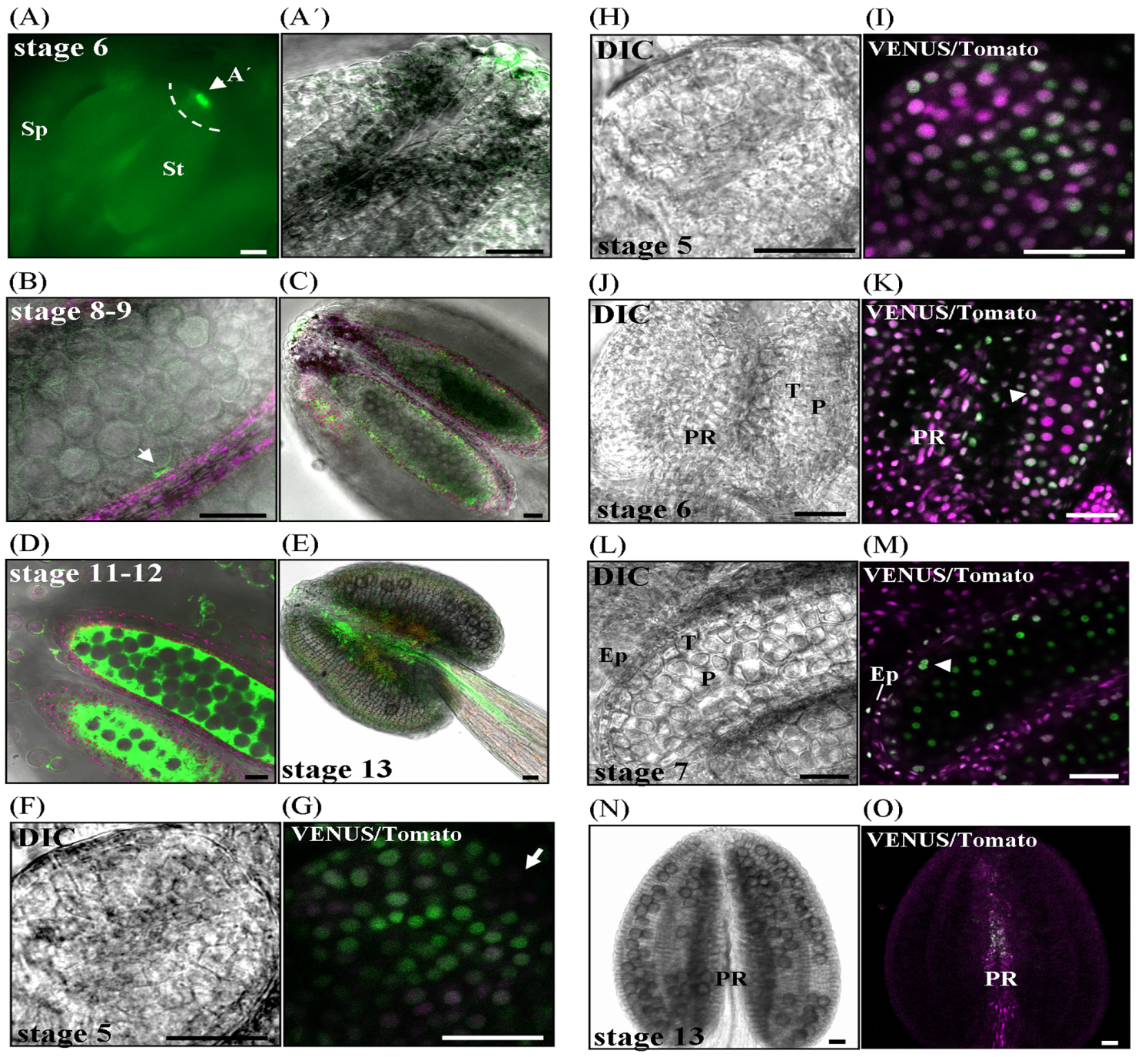
Supplementary Figure 6. Auxin sensing during stamen development. (A-E)** Fluorescence images of *DR5rev:GFP* stamens. **(A)** GFP expression in stage 6 anther. **(A’)** Apical part of the anther delimited in **(A)**. **(B)** White arrow indicates GFP signal in the middle layer. **(A’-E)** are merged GFP/chlorophyll autoflourescence/DIC (green/magenta/grey) pictures obtained using a confocal microscope. **(F-O)** Images of *R2D2* stamens where **(F,H,J,L,N)** are DIC images and **(G,I,K,M,O)** the corresponding Venus/Tomato (green/magenta) overlaid images. **(F-I)** Stack of pictures of a stage 5-anther showing the epidermal cell layer **(F-G)** and the internal cell layers **(H-I).** The white arrow in **(G)** points to the apical part of the anther where fluorescence signal is very low. The white arrowheads in **(K,M)** point to tapetal nuclei. Sp: Sepal, St: Stamen T: tapetum, P: Pre-pollen or pollen, PR: Procambium, EP: Epidermis. Bars = 20 μm.

**
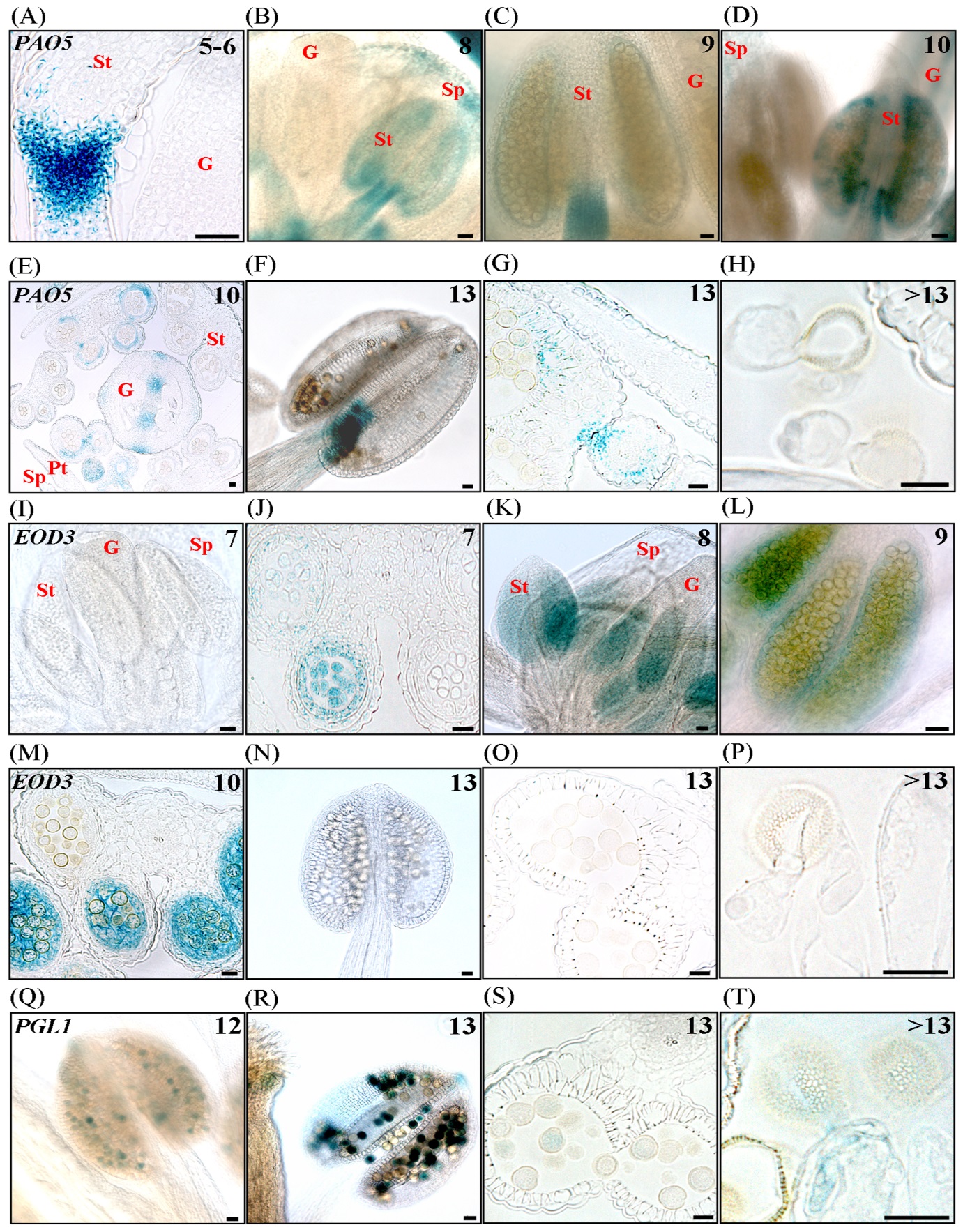
Supplementary Figure 7. *PAO5pro:GUS,* *EOD3pro:GUS* and *PGL1pro:GUS* expression patterns in stamens, analyzed by histochemical GUS assay. (A-H)** *PAO5pro:GUS* staining. **(I-P)** *EOD3pro:GUS* staining. **(Q-T)** *PGL1pro:GUS* staining. (**A,E,G,H,J,M,O,P,S,T**) show cross-sections, while the rest of the figures are whole mount samples of entire floral buds or individual stamens. **(H,P,T)** show germinating pollen on stigmas at anthesis. (**A**) represents anther stage 5-6, (**I-J**) stage 7, (**B,K**) stage 8, (**C,L**) stage 9, (**D,E,M**) stage 10, (**Q**) stage 12 and (**F,G,N,O,R,S**) stage 13. St: Stamen, G: Gynoecium, Sp: Sepal, Pt: Petal. Bars = 20 μm.

## Supplementary Tables

**Supplementary Table1. Oligonucleotides used for cloning and genotyping**

| Oligos for BP cloning strategy into pDONOR221 | | | | |
| --- | --- | --- | --- | --- |
| AGI code | Promoter | | Forward primer (5´🡪3´) | Reverse primer (5´🡪3´) |
| At1g56710 | PGL1 | | attB1-ACTGAAAGCCCATAAGGCCC | attB2-TTGTGAATGTCTTAGGAGATGA |
| At1g19790 | SRS7 | | attB1-TATAAAAGGTGTCTGTTTAACC | attB2-ATTTTTTCTACTTCCCCAACA |
| Oligos for pENTR Directional TOPO cloning strategy | | | | |
| AGI code | Promoter | | Forward primer (5´🡪3´) | Reverse primer (5´🡪3´) |
| At5g12330 | LRP1 | | CACCTAAGAAGAAAAGGTTGGGTAAGTA | AACCCATAAATCTACACAAATCTGA |
| At3g54430 | SRS6 | | CACCTATCCCATCAAAGCTTAA | GTAGTCTGGAGTAGTACTGATTCCA |
| Oligos for genotyping | | | | |
| Name | | Primer (5´🡪3´) | | |
| 021115PGL1.F | | CATCTGATCGTTTGACTTGACA | | |
| 021115SRS6.F | | TTCCCAATGAAGTGGACCACTC | | |
| 021115LRP1.F | | TTAGAGAGAGACAGGAACAAGACG | | |
| 021115SRS7.F | | TCTCTCTCTCTCTCTCACCCACAGA | | |
| 021115pGWB3GUS.R | | CCCACAGGCCGTCGAGTTTT | | |
